# Insomnia and sexual dysfunction associated with severe worsening of the quality of life in sexually active hysterectomized women

**DOI:** 10.5935/1984-0063.20180019

**Published:** 2018

**Authors:** Alvaro Monterrosa-Castro, Angélica Monterrosa-Blanco, Teresa Beltrán-Barrios

**Affiliations:** 1 Grupo de Investigación Salud de la Mujer, Facultad de Medicina. Universidad de Cartagena - Cartagena - Bolívar - Colombia.; 2 Grupo de Investigación Salud de la Mujer, Facultad de Medicina Universidad de las Sabanas. Bogotá. - Bogotá - Cundinamarca - Colombia.

**Keywords:** Menopause, Quality of Life, Insomnia, Sleep, Climacteric, Hysterectomy

## Abstract

Hysterectomy is a common gynecologic surgery carried out to remove the pathologic uterus. **Objective:** To establish if sleep disorders and sexual function are associated with deterioration of the quality of life (QoL) in hysterectomized and sexually active women. **Methods:** A cross-sectional study was carried out with inhabitants from two cities of the Colombian Caribbean. The pollsters invited women aged between 40-59 years to participate; in their communities they applied surveys with demographic characteristics: Female Sexual Function Index, Atenas Insomnia Scale and Menopause Rating Scale. Sexually active women were selected; then the association was established with logistic regression. **Results:** 522 women were studied with an average age of 50 years: 30% oophorectomized, 59.8% Hispanic, 40.2% afro-descendants and 22.2% hormonal therapy users. 80% of them had somato/vegetative, psychological or urogenital deterioration; 29.1% with severe deterioration of QoL and 47.5% with insomnia. Out of 390 (74.7%) with sexual activity, 59.7% suffered from sexual dysfunction. Insomnia: OR:3.05 [95%CI:1.86-4.99], sexual dysfunction OR:3.52 [95%CI:2.01-6.17], dissatisfaction about sexuality OR:4.77 [95%CI:2.08-10.93], low or non-existent sexual desire OR:2.94 [95%CI:1.65-5.25], daytime drowsiness OR:3.15 [95%CI:1.59-6.24] and decrease in daytime well-being OR:3.18 [95%CI:1.79-5.64]. These were factors associated with severe worsening of QoL, while the presence of genital lubrication was protective, OR: 0.44 [95%CI:0.21-0.93], *p*=0.0332. **Conclusion:** It was observed that insomnia and sexual dysfunction behaved as factors associated with three times more severe deterioration of the QoL in climacteric and sexually active women previously hysterectomized.

## INTRODUCTION

Determining the quality of life (QoL) is a priority and it has become an important variable to consider regarding multidisciplinary interest and the approach from different perspectives^1^^,^^2^. QoL includes subjective and objective indicators about physical and psychological well-being, independence, social relationships, environment and spirituality^1^^-^^3^.

The World Health Organization defines the QoL as the perception that the person has about his/her own life within the value system and the cultural context in which they find themselves with respect to their purposes, expectations and concerns^3^. The evaluation should be multidimensional and it has to take into account specific and global components. Several QoL scales have been proposed, which are suitable forms of exploration^1^^,^^3^^,^^4^.

Menopausal symptoms and factors that deteriorate the QoL must be valued during climacteric^5^^-^^9^. Most of the information has been obtained from Caucasian women and the results cannot be systematically extrapolated to other communities or ethnicities. There are no known studies in climacterics previously hysterectomized Colombian women, where the association among sleep, sexuality and QoL is evaluated. The objective was to establish if sleep components and sexual function are associated with worsening of the quality of life (QoL) in hysterectomized and sexually active women.

## METHODS

Cross-sectional study carried out with sociodemographic questions and three validated scales. This investigation is part of CAVIMEC project (Calidad de Vida en la Menopausia y Etnias Colombianas). The project was developed in two phases: a. The properly completed forms to estimate severe deterioration of QoL were involved; b. Sexually active women were chosen to establish the prevalence of sexual dysfunction as well as the association between sexual function and sleep in the QoL. For the evaluation of sexual function and sleep disorders two scales were used: Female Sexual Function Index for the first one and Atenas Insomnia for the second, which are described later.

### Participants

Afro-descendants and Hispanic women aged between 40-59 years old, residing in Cartagena or Barranquilla in the Colombian Caribbean. The pollsters, door by door, identified and invited to participate women with more than 12 months of hysterectomy. Surgically intervened women for malignant or obstetric pathology, vaginal or by laparoscopy and those who did not understand the study were excluded. Only the completed forms were considered.

### Analytic tools

[A] Menopause Rating Scale (MRS): specific to menopausal symptoms and QoL; it is composed by 11 questions that are grouped in three domains: somato/vegetative (hot flashes, cardiac discomfort, sleep disturbance, muscle/joint pain) psychological (depressed mood, irritability, anxiety, physical/mental fatigue) and urogenital (sexual problems, urinary bladder, vaginal dryness). Each question is answered from 0-4 points. The total determines the score of the domain and the total score. Also, the scale identifies four levels of severity: none/little, mild, moderate and severe. Somato/vegetative score greater than 8, psychological greater than 6, urogenital higher than 3 and QoL greater than 16 define severe compromise^4^.

[B] Atenas Insomia Scale (AIS): psychometric instrument about sleep disturbance composed by 8 questions which are answered from 0-3, the sum offers the total score; if is higher than 5, it is a sign of insomnia^10^.

[C] Female Sexual Function Index (FSFI-6): is a one-dimensional abbreviated instrument of a previous proposal. It assesses the sexual function in the last four weeks and identify the sexually active women. Six questions explore desire, arousal, lubrication, orgasm, satisfaction and pain. Each question is marked from 0-5 points. A total score equal to or less than 19 defines sexual dysfunction^11^.

### Sample size

The last Colombian census was carried out in 2005 and 41.468.384 people participated. The estimated impact in 2006 was 48.747.708 inhabitants, 24.678.673 women, 5.660.856 between 40-59 years old, 122.067 residents in Cartagena and 153.008 in Barranquilla; [http://www.dane.gov.co/index.php/estadisticas-por-tema/demografia-y-poblacion/censo-general-2005-1/censo-general-2005]. The study had 384 participants, 50% of heterogeneity level, 5% error and 96% of confidence level. It has been observed in the CAVIMEC study that 30% of the invited women refused to participate in the study due to social, cultural or economic reasons (unpublished data). For the aforementioned condition, 114 women were included and 147 (30%) were added to compensate the incomplete surveys. Therefore, 645 women were involved.

### Statistical analysis

There was created a database with the information from the forms with Microsoft Excel^©^ and analyzed with Epi-Info^©^ and Med-Calc^©^. The quantitative results are expressed in median with interquartile features, the qualitative with percentages 95% CI. The associations were estimated with OR and 95%CI. Additionally, a logistic regression model was carried out with six questions from FSFI-6 and eight from AIS as independent variables. On the other hand, the severe deterioration of QoL was determinated with MRS that was the dependent variable. The stepwise regression was used to identify the proper variables -*p*<0.05 was considered significant-.

### Ethics

The participation was anonymous and voluntary with signed informed consent. This project was endorsed by the Ethics Committee of Universidad de Cartagena, Colombia, according to the rules for research in the health field: Resolución 8430-1993, Ministerio de Salud, República de Colombia [https://www.minsalud.gov.co/sites/rid/Lists/BibliotecaDigital/RIDE/DE/DIJ/RESOLUCION-8430-DE-1993.PDF]. The participants were able to stop filling the form if they considered it pertinent.

## RESULTS

645 women were invited, out of which 22 (3.4%) did not accept to participate. 101 (16.2%) incomplete forms were discarded. In the first phase, 522 women were included, 35.9% above the sample size and the distribution of data was nonparametric. 40.2% was afro-descendants and 59.8% was Hispanic. 68.2% had never smoked with an average age of 50. The 30.0% had bilateral oophorectomy and the 65.3% were hysterectomized during postmenopause. One out of five used hormonal therapy (Table 1).

**Table 1:** Sociodemographic characteristics (n=522).

Age, Me [RI]	50 [46-55]
Number of children, Me [RI]	3 [2-4]
Study years, Me [RI]	10 [7-14]
Weight, Me [RI]	69 [60-78]
Height, Me [RI]	1.6 [1.5-1.6]
Body Mass Index, Me [RI]	26 [23-29]
Marital status single, n (%) [95%CI]	57 (10.9) [8.4-13.9]
Marital status married, n (%) [95%CI]	204 (39.0) [34.9-43.4]
Marital status civil union, n (%) [95%CI]	159 (30.4) [26.5-34.6]
Marital status divorced, n (%) [95%CI]	74 (14.1) [11.3-17.5]
Marital status widow, n (%) [95%CI]	28 (5.3) [3.6-7.7]
Afrodescendant ethnic group, n (%) [95%CI]	210 (40.2) [36.2-44.5]
Mestizo ethnic group, n (%) [95%CI]	312 (59.8) [55.2-64.2]
Number of coffee cups per day, Me [RI]	2 [1-3]
Coffee consumption, n (%) [95%CI]	337 (64.5) [60.2-68.6]
No coffee consumption, n (%) [95%CI]	185 (35.4) [31.3-39.7]
Never smokers, n (%) [IC95%]	356 (68.2) [63.9-72.1]
Previously smokers, n (%) [95%CI]	124 (23.7) [20.2-27.6]
Currently smokers, n (%) [95%CI]	42 (8.0) [5.9-10.8]
Bilateral oophorectomy, n (%) [95%CI]	157 (30.0) [26.2-34.2]
Women with one ovary, n (%) [95%CI]	148 (28.3) [24.5-32.4]
Women with both ovaries, n (%) [95%CI]	217 (41.5) [37.3-45.9]
Last menstruation before surgery, n (%) [95%CI]	341 (65.3) [61.0-69.3]
Menstruation at the time of surgery, n (%) [95%CI]	181 (34.6) [30.6-38.9]
Hormone therapy users, n (%) [95%CI]	116 (22.2) [18.7-26.0]
Non-users of hormone therapy, n (%) [95%CI]	406 (77.7) [73.9-81.2]

The 19.1% showed severe/very severe hot flashes. Eight out of ten experimented in some grade somato/vegetative, psychological, urogenital or QoL deterioration. Half had severe urogenital deterioration, while two out of ten experienced severe psychological worsening. The domain with minor severe compromise was the somato/vegetative (Table 2). Severe deterioration of QoL was observed in 152 (29.1%) [95%CI:25.2-33.2]. In contrast, 274 (52.4%) [95%CI:66.7-74.1] women did not present it, *p*<0.0001.

**Table 2:** Menopause rating scale (*) deterioration of domains and quality of life (n=522).

	None/Little n (%) [95%CI]	Mildn (%)[95%CI]	Moderate n (%) [95%CI]	Severe n (%) [95%CI]
Somato-vegetative	124 (23.7) [20.2-27.6]	133 (25.4) [21.8-29.4]	191 (36.5) [32.4-40.9]	74 (14.1) [11.3-17.5]
Psychological	114 (21.8) [18.4-25.6]	193 (36.9) [32.8-41.2]	112 (21.4) [18.0-25.2]	103 (19.7) [16.4-23.4]
Urogenital	97 (18.5) [15.3-22.2]	60 (11.4) [8.9-14.6]	109 (20.8) [17.5-24.6]	256 (49.4) [44.6-53.4]
Quality of life	85 (16,2) [13.2-19.8]	95 (18.0) [14.8-21.6]	190 (36.4) [32.2-40.7]	152 (29.1) [25.4-33.4]

Table 3 presents the findings with AIS. The 68.8% considered a problem to wake up in the middle of the night and the 56.4% had problems with the induction of sleep. For half of the women awakening hours were earlier, insufficient duration of sleep, decreased physical/mental functioning, sleep quality was insufficient or the well-being decreased during the day. 51.3% did not present daytime drowsiness, slight 33.5%, considerable 11.6% and intense 3.4%. It was found that 248 (47.5%) [95% CI: 43.1-51.8] had insomnia and 274 (52.4%) [95% CI: 48.1-56.8] did not present it, *p*=0.2.

**Table 3:** Atenas Insomnia Scale (*) (n=522).n (%) [95%CI].

Sleep induction	Any problem	Mild Problema	Moderate Problema	Delayed
	228 (43.6) [39.3-48.0]	212 (40.6) [36.3-44.9]	66 (12.6) [9.9-15.8]	16 (3.0) [1.8-5.0]
Wake up at night	Any problem	Mild problem	Considerable Problema	Serious problem
	163 (31.2) [27.3-35.4]	270 (51.2) [47.3-56.0]	76 (14.5) [117-17.9]	13 (2.4) [1.3-4.3]
Wake up earlier	No more	A Little	Markedly	Much more
	232 (44,4) [40.1-48.8]	223 (42.7) [38.4-47.1]	54 (10.3) [7.9-13.3]	13 (2.4) [1.3-4.3]
Total sleep duration	Suficient	Slightly insufficient	Markedly insufficient	Very insufficient
	254 (48.6) [44.3-53.0]	195 (37.6) [33.2-41.6]	60 (11,4) [8.9-14.6]	13 (2.4) [1.3-4.3]
General quality of the sleep	Satisfactory	Slightly insufficient	Moderately insufficient	Very insufficient
	263 (50.3) [46.0-54.7]	172 (32.9) [28.9-37.1]	75 (14.3) [11.5-17.7]	12 (2.3) [1.2-4.0]
Feeling of well-being during the day	Normal	Slightly diminished	Marckedly diminished	Very diminished
	267 (51.1) [46.7-55.5]	197 (37.7) [33.5-42.0]	40 (7.6) [5,6-10,3]	18 (3.4) [2.1-5.5]
Physical-mental functioning during the day	Normal	Slightly diminished	Marckedly diminished	Very diminished
	273 (52.3) [47.9-56.6]	190 (36.4) [32.2-40.7]	49 (9.3) [7.0-12.3]	10 (1.9) [0.9-3.6]
Drowsiness during the day	None	Mild	Considerably	Intense
	268 (51.3) [46.9-55.7]	175 (33.5) [29.5-37.7]	61 (11,6) [9.1-14.8]	18 (3.4) [2.1-5.5]

55.1% of the women had low/no sexual desire, 29.3% moderate and 15.5% high/very high. 390 (74.7%) reported having sexual activity and make up the sexually active group. Among them, two out of ten considered high/very high their level of arousal. 38.4% never, almost never or only sometimes presented genital lubrication. Less than 12.0% reported having orgasm almost always. The 21.2% declared themselves satisfied with their sex life, while three out of ten were considered unsatisfied. The 23.3% had coital pain sometimes, 14.8% most of the time and 4.8% almost always (Table 4). Sexual dysfunction was estimated to be present in 233 (59.7%) [95% CI: 54.6-64.6], while 157 (40.3%) [95% CI: 35.3-45.3] did not present it, *p*<0.0001.

**Table 4:** Abbreviated index of female sexuale function (*). All women involved n=522

Desire	Very high	High	Moderate	Low	Non-existent
n (%) [95%CI]	30 (5.7) [3.9-8.2]	51 (9.7) [7.4-12.7]	153 (29.3) [25.4-33.4]	149 (28.5) [24.7-32.6]	139 (26.6) [22.9-30.6]
Women with sexual activity in the last four weeks n=390 (74.7%)					
Desire n (%) [95%CI]	Very high	High	Moderate	Low	Non-existent
	30 (7.6) [5.3-10.9]	51 (13.0) [9.9-16.9]	150 (38.4) [33.6-43.5]	119 (30.5) [26.0-35.3]	40 (10.2) [7.5-13.8]
Arousal n (%) [95%CI]	Very high	High	Moderate	Low	Non-existent
	35 (8.9) [6.4-12.3]	55 (14.1) [10.8-18.0]	146 (37.4) [32.6-42.4]	112 (28.7) [24.3-33.5]	42 (10.7) [7.9-14.3]
Lubrication n (%) [95%CI]	Almostalways	Mosttimes	Sometimes	Sometimes	Almost never or never
	25 (6.4) [4.2-9.4]	61 (15.6) [12.2-19.7]	154 (39.4) [34.6-44.5]	95 (24.3) [20.2-28.9]	55 (14.1) [10.8-18.0]
Orgasm n (%) [95%CI]	Almost always	Mosttimes	Sometimes	Sometimes	Almost never or never
	45 (11.5) [8.6-15.2]	93 (23.8) [19.7-28.4]	84 (21.5) [17.6-26.0]	101 (25.9) [21.6-30.6]	67 (17.1) [13.6-21.3]
Satisfaction n (%) [95%CI]	Very satisfied	Moderate	Identical	Moderate dissatisfaction	Very dissatisfied
	83 (21.2) [17.3-25.7]	101 (25.9) [21.6-30.6]	74 (18.9) [15.2-23.3]	84 (21.5) [17.6-26.0]	48 (12.3) [9.3-16.0]
Pain n (%) [95%CI]	Almost never or never	Rarely	Sometimes	Most times	Almost always or always
	147 (37.6) [32.9-42.7]	75 (19.2) [15.5-23.5]	91 (23.3) [19.2-27.9]	58 (14.8) [11.5-18.8]	19 (4.8) [3.0-7.6]

Sexual dysfunction and insomnia were associated three times with increased possibility of severe deterioration of QoL in sexually active hysterectomized women, *p*<0.0001 (Table 5). According to the adjusted logistic regression model: being unsatisfied with the sexuality, decreased sense of well-being during the day, daytime drowsiness and low/no sexual desire were factors significantly associated with severe deterioration of the QoL. In turn, having almost always, most of sometimes or sometimes genital lubrication was a protective factor, *p*=0.03 (Table 6).

**Table 5:** Severe deterioration of the quality of life according to the presence of sexual dysfunction or insomnia in women with current sexual activity (n=390).

		Severe deterioration of the quality of lifeN (%) [95%CI]		OR [95%CI]		*p*
		Yes	No	Not adjusted	Adjusted	
Sexual dysfunction	Yes	105 (83.3) [75.6-89.3]	128 (48.4) [42.3-54.6]	5.5 [3.13-8.99]	3.52 [2.01-6.17]	<0.0001
	No	21 (16.6) [10.6-24.3]	136 (51.5) [45.3-57.6]			
Insomnia	Yes	89 (49.4) [41.9-56.9]	91 (50.5) [43,0-58,0]	4.57 [2.88-7.24]	3.05 [1.86-4.99]	<0.0001
	No	37 (17.6) [12.7-23.4]	173 (82.3) [76.5-87.2]			

**Table 6:** Factors associated with severe deterioration of the quality of life adjusted logistic regression.

	Coefficient	Standard error	*p*	OR	95%CI
Insatisfaction about sexuality	1.5624	0.4233	0.0002	4.77	2.08-10.93
Decreased sense of well-being during the day	1.1574	0.2922	0.0001	3.18	1.79-5.64
Daytime drowsiness	1.1486	0.3486	0.0010	3.15	1.59-6.24
Low/non-existent sexual desire	1.0797	0.2954	0.0003	2.94	1.65-5.25
Genital lubrication presence	-0.8066	0.3788	0.0332	0.44	0.21-0.93
CONSTANT	-2.7981				

Excluded variables in the model according to stepwise regression: sleep induction, wake up at night, wake up earlier, sleep duration, sleep quality, physical-mental functioning during the day, excitement, orgasm and coital pain. Chi-squared: 110.62; DF: 5; *p*>0.1.

## DISCUSSION

### Hysterectomy

Is a surgical intervention commonly indicated to treat myomas, severe endometriosis, chronic pelvic pain, gynecological masses and uterine hemorrhages^2^^,^^9^. In the last decades women have achieved better conditions inside the productive structure of the society, managing positions that demand for them to take decisions and different responsibilities. For this social positioning women also require better health conditions and be free of abnormal genital bleeding, anemia, colic or cyclical pelvic discomfort that deteriorate their QoL^2^. The hysterectomy reduces those symptoms, for that reason it is perceived as beneficial due to the improvement of QoL^2^^,^^11^.

For some communities, the uterus symbolizes femininity and sexual value, so the hysterectomized woman loses hierarchy, is vulnerable to her partner, and becomes susceptible to social signaling for the detriment of QoL^12^^,^^13^. Moreover, the hysterectomy can cause new symptoms due to the reduced availability of ovarian hormones, as well as vaginal shortening with involvement of the QoL^9^. As a result, women have to be informed about the expected benefits, potential somatic, psychological, urogenital impairment and consequences in the QoL because of the intervention. The aforementioned consequences are influenced by personal (ethnicity, age, biological conditions), social, educational and sanitary aspects^14^^,^^15^.

It was observed that four out of five middle-aged women had, in some grade, deterioration of those domains and the QoL. Half of them had severe urogenital deterioration and three out of ten had severe deterioration of QoL. Saavedra-Orozco et al.^15^ found a similar clinical profile in other group of hysterectomized Colombian women evaluated with MRS: 18.7% presented severe/very severe hot flashes and 25.7% severe/very severe sexual problems. These figures are high and show the need for health interventions.

In the United States, 600,000 hysterectomies are performed annually and half of them include bilateral oophorectomy^16^. One third of the participants had bilateral oophorectomy in this study. Deciding to remove the ovaries with the uterus must be a decision supported by clinical criteria, since the removal of the gonads causes immediate loss of estradiol, testosterone, and androstenedione^13^^,^^16^. Finch et al.^17^ found greater vasomotor, psychological, physical and sexual deterioration after bilateral oophorectomy compared with preoperative evaluation, which showed that surgical menopause affects negatively the health and QoL due to hypoestrogenism and hypoandrogenism.

As mentioned earlier, hormonal cessation is associated with an increase of 50% in the risk of osteoporotic fracture, double increase in mortality after hip fracture caused by trauma and double risk of Parkinson disease and cognitive deterioration^2^^,^^5^. The Women´s Health Initiative^18^ informed that hysterectomy with oophorectomy is an independent factor for myocardial infarction or death by coronary heart disease.

The hysterectomy with oophorectomy can trigger sudden onset of hot flashes, mood changes and vaginal atrophy, which are related to damage of the QoL and sexual deterioration^17^. Oophorectomized women had worse sexual function than those not oophorectomized, both in sexual desire, frequency and orgasmic response^16^.

### Sexual function

Several authors^8^^,^^19^^,^^20^ have indicated that the greatest sexual deterioration in women undergoing surgical menopause is due to the reduction of estradiol and testosterone. The Women´s International Study of Health and Sexuality^21^ reported that women aged between 20-49 years and with surgically induced menopause had higher rates of hypoactive sexual desire disorders compared with those who had intact gonads: 26% versus 14% respectively, *p*<0.0001, which coincides with the study findings where dysfunction, low desire and dissatisfaction were risk factors for deterioration of QoL, *p*<0.0001.

The appropriate vaginal health is important for sexuality^20^. The decrease of estrogen is associated with genitourinary atrophy, lactobacilli reduction, basic pH, changes in flora and vaginal dryness^19^^,^^22^. Women will experiment pain during sexual activity, lose interest and avoid sexual relationships. All of the previous have negative impact on their QoL^23^. Half of women with genitourinary atrophy inform that the symptoms interfere with the sexual enjoyment; 12% of the women without a couple prefer to be alone due to these symptoms^6^^,^^24^.

In the Vaginal Health: Insights, Views & Attitudes study^7^, 75% of women informed that vaginal discomfort negatively affected their sexual life. Vaginal dryness induces worse emotional well-being and social functioning^5^. The presence of adequate lubrication was a protective factor for deterioration of QoL in the Colombian studied women, *p*=0.03.

Many hysterectomized women can be sexually active, if they have a stable couple. Although most of them consider that sex is an important part of life, as was observed in the study, dissatisfaction and sexual dysfunction are highly prevalent^8^. Generally, few women reveal their sexual concerns, hence the importance of medical thoroughness. If sexual dysfunction is suspected, the doctor must deepen into social and medical history with questions focused on sexuality. Discovering the etiology and identifying modifiable factors can lead to improvements in the sexuality and QoL^20^. Sexual dysfunction and QoL are multidimensional concepts, interrelated in both the reproductive and climacteric stages^25^.

The perception, participation and expectations of the sexual partner, before or after hysterectomy, are poorly studied. Solidarity and empathy between the couple can reduce false expectations or preventions regarding surgery. Men tend to worry about hysterectomy impact, especially with regard to sexuality. Both, man and woman, should receive complete and precise information, always taking into account cultural customs and sexual behaviors^2^.

### Sleep disorders

Are frequent alterations in postmenopausal women. They can be primary or secondary to hot flashes, disorders of the state of sleep, medical conditions, psychosocial factors or intrinsic sleep disorders that, if remain untreated, can cause deterioration of the QoL. Insomnia, well-being diminution and daytime drowsiness were factors associated with poor QoL among the Colombians women analyzed in this study. It has been showed that ovarian hormones have favorable impact in the sleep^26^.

Progesterone stimulates benzodiazepine receptors with sedative and anxiolytic effect^27^. Estrogen has a benefit impact on sleep architecture; also, it is involved in the metabolism of norepinephrine, serotonin and acetylcholine: neurotransmitters that control sleep, increase the total time of sleep and diminish the sleep latency and the number of awakenings^28^. Memory loss, a recurrent complain among postmenopausal women, can be associated with poor sleep architecture^29^.

Having said that, the average user of hormonal therapy had fewer night awakenings than non-user in a study with 3000 postmenopausal women^30^. It is difficult to identify the beneficial effect of estrogen compared to progesterone. Both hormones seem to have independent therapeutic effects on sleep^25^^,^^31^. Testosterone and sleep have not been extensively studied; apparently, they do not alter normal sleep^32^. However, the etiology of sleep disorders is multifactorial; it must be evaluated and treated because it goes deeper than menopause. The etiology may arise as part of aging and it is not necessarily related to the decrease of estrogen levels.

### Limitations

This research has typical limitations of cross-sectional studies. There was not evaluation of sexual dysfunction, nor daytime insomnia or sleepiness, or of QoL before the surgery. It is necessary to carried out mores studies with other designs that allow a greater approach to causal relationships among sexual dysfunction, sleep disorders and QoL in hysterectomized women. Although only women operated for benign pathology were included, the diagnosis that motivated the surgery, complications or subsequent evolution was not specified.

The time from the intervention to the subsequent evolution was not establish in order to determine if QoL and the identified factors are modified with the time. In spite of the results cannot be overtly extrapolated, they are the first approaches to the study of QoL, sleep disorders and the sexuality of hysterectomized climacteric women in Latin America and the Colombian Caribbean. On the other hand, it has as a strength that it was carried out in the community with tools that measure the perception of women. The tools are widely validated and had good statistical reliability in the population involved.

### Recommendations

Sleep and sexuality disorders must be evaluated and treated in hysterectomized women. It is necessary to take into account biological, ethnic, cultural, environmental and familiar aspects.

## CONCLUSION

It was observed that insomnia and sexual dysfunction behaved as factors associated with three times more severe deterioration of the QoL in climacteric and sexually active women previously hysterectomized.
